# The Role of Digital Health Equity Audits in Preventing Harmful Infodemiology

**DOI:** 10.2196/75495

**Published:** 2025-05-30

**Authors:** Massimiliano Biondi, Fabio Filippetti, Giorgio Brandi, Elsa Ravaglia, Sofia Filippetti, Pamela Barbadoro

**Affiliations:** 1 Medical Directorate, Fabriano Hospital Site, World Federation of Public Health Associations GHEDT WG, Ancona Health Authority AST AN, Via Stelluti Scala, 26, Fabriano, 60044, Italy, 39 0732 707111; 2 Prevention and Health Promotion Unit in Living and Working Places of the Marche Region, Ancona, Italy; 3 Unit of Hygiene, Department of Biomedical Sciences, University of Urbino Carlo Bo, Urbino, Italy; 4 Pesaro-Urbino Health Authority AST PU, Pesaro, Italy; 5 Department of Public Health and Pediatrics, University of Turin, Turin, Italy; 6 Unit of Hygiene, Preventive Medicine and Public Health, Department of Biomedical Sciences and Public Health, Faculty of Medicine, Marche Polytechnic University, Ancona, Italy

**Keywords:** equity, digital, audit, infodemiology, quality of health care

## Abstract

**Background:**

Health disparities persist and are influenced by digital transformation. Although digital tools offer opportunities, they can also exacerbate existing inequalities, a problem amplified by the COVID-19 pandemic and the related infodemic. Health equity audit (HEA) tools, such as those developed in the United Kingdom, provide a framework to assess equity but require adaptation for the digital context. Digital determinants of health (DDoH) are increasingly recognized as crucial factors influencing health outcomes in the digital era.

**Objective:**

This editorial proposes an approach to extend HEA principles to create a specific framework, the digital health equity audit (DHEA), designed to systematically assess and address health inequities within the design, implementation, and evaluation of digital health technologies, with a focus on DDoH.

**Methods:**

We propose a cyclical DHEA model based on existing HEA principles, integrating them with digital health equity frameworks. The DHEA cycle comprises six phases: (1) scoping the audit and mobilizing the team (including community members); (2) developing the digital health equity profile and identifying inequities (assessing DDoH at individual, interpersonal, community, and societal levels); (3) identifying high-impact actions to address DDoH and inequities; (4) prioritizing actions for maximum equity impact; (5) implementing and supporting change; and (6) evaluating progress and impact, and refining. This method emphasizes multilevel interventions and stakeholder engagement.

**Results:**

The main result is the articulation of the DHEA framework: a structured, 6-phase cyclical model to guide organizations in the analysis and proactive mitigation of digital health–related disparities. The framework explicitly integrates the assessment of DDoH across multiple levels (individual, interpersonal, community, societal) and promotes the development of targeted interventions to ensure digital solutions promote equity.

**Conclusions:**

The DHEA model offers an integrated approach to consider social, epidemiological, health, and technological variables, aiming to reduce health inequities through the conscious use of new technologies. It is emphasized that digital technologies can be the cause or the solution to inequalities; DHEAs are proposed as a tool to foster equity. Its systematic adoption, along with a collaborative approach (co-design) and trust building, can help ensure that the benefits of health digitization are equitably distributed while strengthening trust in institutions. Continued attention is needed to manage emerging challenges such as infodemiology in the era of big data and artificial intelligence.

## Introduction

The concept of a health equity audit (HEA), as part of guidance issued by Public Health England (PHE) in the context of their Health Equity Assessment Tool, updated in May 2021, helps to articulate a clear framework to address health inequalities and has potential to be extended to the broader field of digital health [1]. Specifically, the PHE guidance defined how the objective of HEAs is to evaluate whether resources are distributed equitably with respect to the health needs of different population groups; they systematically examine health inequalities and access to services for particular groups or areas. Audits also ensure that actions to address health inequalities are incorporated into planning decisions, prioritizing actions to address health inequalities, and addressing how they can evaluate the impact of the actions on reducing inequalities.

These tools, despite having been widely used in the United Kingdom since the 2000s and subsequently neglected due to organizational changes in the British health care system, are currently recommended by PHE, which following the inequalities evidenced during the COVID-19 pandemic, renewed interest in adopting the tool for health care purposes; the tool could also be adapted for various applications of digital health technologies [2,3].

As the field of health disparities has matured to reach a crucial element of health care management and quality, we have simultaneously witnessed the effects of digital transformation on health status as well as health care [4-7]. This necessitates the need for a reexamination of the utility of HEAs in the design, implementation, and evaluation of digital health technologies. These inequalities have amplified during the COVID-19 pandemic, especially due to the misinformation caused by the infodemic, which pushed the World Health Organization to call a conference on the topic. Importantly, the convergence of factors such as volume and speed of information, misinformation, and disinformation flow, combined with political polarization, requires the forging of a community for the evidence-based practice of infodemic management [8].

## Different Definitions of Digital Health

According to the National Institutes of Health, digital health refers to the use of information and communication technologies in medicine and other health professions to manage diseases and health risks and promote well-being [9]. For the European Union, digital health and care refers to tools and services that use information and communication technologies to improve the prevention, diagnosis, treatment, monitoring, and management of health-related problems, and to monitor and manage lifestyle habits that affect health. Digital health and care facilitates the use of emerging and innovative technologies, and has the potential to improve access and the quality of care, as well as increase the overall efficiency of the health care sector [10].

The World Health Organization [11] has three key objectives to promote the adoption and expansion of digital health and innovation:

1. Promote data sharing and support the implementation of digital solutions that contribute to informed decision-making
2. Improve knowledge through the best scientific communities
3. Assess and connect countries’ needs with the supply of innovations

Access to digital technologies in health, including the internet, technological tools, digital agendas and systems, digital literacy, etc, has also become an increasingly important determinant of health and has a special relationship with social determinants of health. Emerging evidence from the scientific literature recognizes that access to digital technologies is now a determinant of health outcomes [12,13]. As digital determinants of health (DDoH) become increasingly recognized [14,15], a framework for digital health equity audits (DHEAs), including the evaluation of key DDoH, is needed.

## Opportunities to Extend HEA Tools

In this editorial, we propose an approach to extend HEA tools to address shared international objectives of synergistically promoting both health equity and digital health adoption and access, framed as a DHEA. This strategy is rooted in the World Health Organization’s objectives of equity and digitalization as described above and involves the synthesis of a tool that combines HEA concepts with stated goals of digital health equity, as published in other academic literature, and modeled based on proposals by the Agency for Healthcare Research and Quality in a comprehensive framework [16]. We support the implementation of a similar approach also focusing on improving the health status of the population in relation to the use of technologies and the context of health technology assessments. However, we must consider that new digital “approaches have the potential to address some of the structural challenges for marginalized populations.... Yet the digitalization of health care can also harm health equity if this digitally enabled ecosystem moves forward without proactive engagement, planning, and implementation” [5]. As discussed in the recent literature, the evidence linking inequality in health care to misinformation exposure and mitigating strategies is a complex area where further research is needed [17-19].

## The DHEA Cycle

### Overview

Building upon standard HEA principles, the DHEA cycle integrates the Framework for Digital Health Equity to specifically address how digital technologies impact health disparities. It emphasizes understanding and acting on DDoH across multiple levels (individual, interpersonal, community, societal) to ensure digital health solutions promote equity rather than widen gaps.

**Figure 1.**
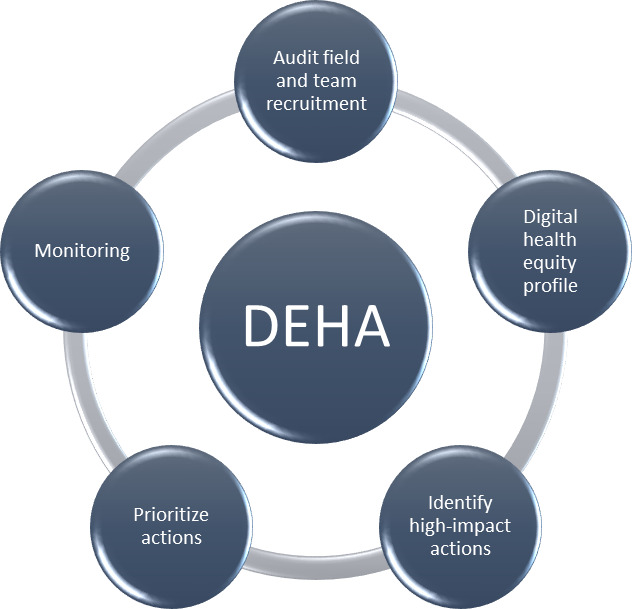
Digital health equity audit (DHEA).

The DHEA cycle phases are shown in Figure 1.

### Scope the Audit and Mobilize the Team (Phase 1)

#### Action

A diverse working group is formed including community members (especially from disparity populations), clinicians, IT specialists, designers, policy makers, and public health professionals.

#### Integration

The specific digital health tool, service, or system being audited (eg, a new patient portal feature, a telehealth service) is defined. Priority populations and potential equity concerns are agreed on, explicitly considering the Framework for Digital Health Equity [15] and its emphasis on populations adversely affected by health differences (racial/ethnic minorities, those with low-income, those who live in rural areas, sexual and gender minorities, or individuals with disabilities).

#### Example

When auditing a new telehealth platform for primary care, the team can include patient representatives from low-income neighborhoods, accessibility experts, primary care physicians, and IT developers, prioritizing equitable access and usability for seniors and nonnative speakers.

### Develop the Digital Health Equity Profile and Identify Inequities (Phase 2)

#### Action

Data is gathered to create a profile of the target population’s interaction with the specific digital health tool/service and the broader digital environment, using surveys, interviews, use data, population health data, and community assessments.

#### Integration

This profile must assess relevant DDoH across the four levels:

- Individual: digital literacy, technology access (device/internet), digital self-efficacy, attitudes/trust toward technology
- Interpersonal: implicit technology bias from providers, patient-provider communication via digital tools, family/caregiver technology interdependence
- Community: broadband availability/affordability, local technology support resources (libraries, community centers), health care system’s digital infrastructure quality, relevant community technology norms
- Societal: technology policies (reimbursement, privacy), data/design standards (accessibility, language), algorithmic bias, social norms around technology

#### Action Continued

The profile is analyzed to pinpoint specific inequities—where are there avoidable unfair differences in digital access, use, experience, or outcomes between population groups?

#### Example

The profile for the telehealth platform reveals that seniors have lower adoption rates. An analysis identifies key DDoH barriers: lower digital literacy and lack of affordable broadband (individual/community), coupled with clinician assumptions about seniors’ ability/interest (interpersonal implicit technology bias). An inequity is identified: seniors face avoidable barriers to accessing telehealth compared to younger higher-income groups.

### Identify High-Impact Actions to Address DDoH and Inequities (Phase 3)

#### Action

Potential interventions are suggested to address the specific DDoH barriers and inequities identified in phase 2, reviewing the evidence for effective strategies.

#### Integration

The focus is on developing multilevel interventions that target “upstream” determinants (community and societal levels) where possible, as these often have a broader and more sustainable impact on equity, as highlighted by the summarized text. Actions addressing individual skills, interpersonal interactions, community resources, and systemic policies/design should be considered.

#### Example

The following actions can be taken to address the telehealth inequity:

- Individual: offer digital literacy training tailored for seniors; provide loaner tablets
- Interpersonal: train clinicians on identifying and mitigating implicit technology bias
- Community: partner with local libraries or senior centers for technology support hubs; advocate for expanded community broadband initiatives
- Societal: Advocate for policies ensuring telehealth platforms meet high accessibility standards (Web Content Accessibility Guidelines)

### Prioritize Actions for Maximum Equity Impact (Phase 4)

#### Action

The potential actions are evaluated based on criteria such as potential impact on reducing the identified inequity, feasibility, cost, community acceptability, and alignment with organizational goals.

#### Integration

Actions most likely to address root causes (upstream DDoH) and benefit populations disproportionately are prioritized, ensuring the prioritization process involves stakeholders, especially from affected communities.

#### Example

Partnering with senior centers for training/support (community/individual: high impact, feasible) and advocating for better broadband (community/societal: high upstream impact, longer term) can be prioritized over simply providing tablets without support (individual: less sustainable).

### Implement and Support Change (Phase 5)

#### Action

An implementation plan is developed, allocating necessary resources (funding, staffing, partnerships) and executing the prioritized actions.

#### Integration

Resources need to specifically address the DDoH barriers (eg, funding for digital navigators, accessible design implementation, community infrastructure partnerships). Synergy needs to be fostered between the implementation team, decision makers, and the target community through ongoing communication and feedback loops, adapting based on initial rollout experiences.

#### Example

The following actions can be taken during this phase: secure funding for trainers at senior centers, deploy accessible platform updates, launch clinician training modules, or establish a feedback channel with senior users.

### Evaluate Progress and Impact, and Refine (Phase 6)

#### Action

The implementation process is monitored, and the impact of the actions is evaluated against the initial objectives and the identified inequities.

#### Integration

Specific, measurable indicators are defined that track changes in DDoH (eg, digital literacy scores, broadband access rates, device ownership) and health equity outcomes related to the digital tool (eg, telehealth use rates stratified by age/income/race, patient satisfaction scores by demographic group, changes in relevant health metrics for disparity groups). Evaluation findings need to be used to refine actions, inform future DHEA cycles, and demonstrate accountability.

#### Example

Telehealth appointment completion rates can be tracked to analyze seniors versus other groups, pre- and postscores for the digital literacy assessment for participants, qualitative feedback on usability, and number of broadband sign-ups through advocacy efforts. If senior use remains low, phases 2 and 3 can be revisited to identify potentially missed DDoH barriers.

## Conclusions

The DHEA model is an integrated model that takes into account social, epidemiological, health, and technological variables. The integration of knowledge and resources, together with the involvement at the institutional and the population levels, should produce a health gain for the majority of the population, reducing health inequities thanks to new technologies and strengthening trust in government institutions and health care. The systematic adoption of integrated and digitized tools for reading the system could certainly contribute to the evaluation of the effectiveness of interventions [20]. However, the digitization of health services in 2025 is increasingly important and its massive implementation is expected in the following years. The large amount of data that we will have to manage—fueled by the continuous flow of information—is converging and will converge with artificial intelligence all over the world, leading to a rapid and proportionally difficult-to-control diffusion of infodemiology. The increased convenience, accessibility, and penetration of internet services have significantly transformed how people obtain information on health-related issues. The rapid proliferation of information and communication technology tools has led to an era of unprecedented accessibility to vast repositories of information, especially through online communication channels and social media platforms [21]. In summary, we could therefore affirm that new technologies can be the cause of inequalities or the solution to health inequalities. We would like the DHEA tool to help increase equity at all levels so that one day everyone can benefit from the advantages of technologies and, through them, be in control of their health and well-being. Facilitators include building trust (eg, providing evidence for health messages), while barriers include user reluctance to accept support. The main recommendations are adopting a collaborative working approach (involving users, developers, health care professionals, policy makers, etc, often through co-design) and using effective advertising to raise awareness of available support.

Being aware of the great advantages of the widespread, adequate, and fair use of continuous technological and digital innovations available to science, we must never forget that they are the tool and not the goal.

## Abbreviations

DDoH: digital determinants of health
DHEA: digital health equity audit
HEA: health equity audit
PHE: Public Health England
